# Targeted thorium-227 conjugates as treatment options in oncology

**DOI:** 10.3389/fmed.2022.1071086

**Published:** 2023-01-09

**Authors:** Jenny Karlsson, Christoph A. Schatz, Antje M. Wengner, Stefanie Hammer, Arne Scholz, Alan Cuthbertson, Volker Wagner, Hartwig Hennekes, Vicki Jardine, Urs B. Hagemann

**Affiliations:** ^1^Bayer AS, Oslo, Norway; ^2^Bayer AG, Berlin, Germany; ^3^Bayer Consumer Care AG, Basel, Switzerland; ^4^Bayer PLC, Reading, United Kingdom

**Keywords:** alpha-particle emitter, DNA damage response, immune checkpoint inhibitors, anti-androgen therapies, alpha emitter, targeted alpha therapy, targeted thorium conjugates, thorium-227

## Abstract

Targeted alpha therapy (TAT) is a promising approach for addressing unmet needs in oncology. Inherent properties make α-emitting radionuclides well suited to cancer therapy, including high linear energy transfer (LET), penetration range of 2–10 cell layers, induction of complex double-stranded DNA breaks, and immune-stimulatory effects. Several alpha radionuclides, including radium-223 (^223^Ra), actinium-225 (^225^Ac), and thorium-227 (^227^Th), have been investigated. Conjugation of tumor targeting modalities, such as antibodies and small molecules, with a chelator moiety and subsequent radiolabeling with α-emitters enables specific delivery of cytotoxic payloads to different tumor types. ^223^Ra dichloride, approved for the treatment of patients with metastatic castration-resistant prostate cancer (mCRPC) with bone-metastatic disease and no visceral metastasis, is the only approved and commercialized alpha therapy. However, ^223^Ra dichloride cannot currently be complexed to targeting moieties. In contrast to ^223^Ra, ^227^Th may be readily chelated, which allows radiolabeling of tumor targeting moieties to produce targeted thorium conjugates (TTCs), facilitating delivery to a broad range of tumors. TTCs have shown promise in pre-clinical studies across a range of tumor-cell expressing antigens. A clinical study in hematological malignancy targeting CD22 has demonstrated early signs of activity. Furthermore, pre-clinical studies show additive or synergistic effects when TTCs are combined with established anti-cancer therapies, for example androgen receptor inhibitors (ARI), DNA damage response inhibitors such as poly (ADP)-ribose polymerase inhibitors or ataxia telangiectasia and Rad3-related kinase inhibitors, as well as immune checkpoint inhibitors.

## 1. Introduction

Despite drug discovery advances, an unmet clinical need for novel oncology treatment modalities persists. Targeted alpha therapy (TAT) represents one such modality, as α-particles have several properties of potential value in cancer therapy. These include high linear energy transfer (LET), short penetration range, and induction of complex double-stranded DNA breaks (1). High LET means a low number of hits are needed to induce cell death (1), while the short-path length of α-particles (50–100 μm) is expected to minimize damage to surrounding healthy tissue (1). Furthermore, complex double-stranded DNA breaks induced by alpha-radiation are hard to repair, promoting cell cycle arrest and cell death (1, 2). TATs may also promote T-cell infiltration through induction of immunogenic cell death (3–6), or have increased potency against tumor cells with alterations in DNA damage repair genes (cytotoxic radiation-induced DNA damage increases their susceptibility to apoptosis) (7–9).

Selective tumor targeting by TATs can be achieved through two primary mechanisms: inherent radionuclide properties (1) and the ability to chelate the radionuclide to a tumor-targeting molecule (e.g., a monoclonal antibody, peptide or small molecule) (1). Over the last 20 years, several α-particle-emitting radionuclides have been investigated as TATs, including: actinium-225 (^225^Ac, half-life 9.9 days); astatine-211 (^211^At, half-life 7.2 h); bismuth-213 (^213^Bi, half-life 45.6 min); radium-223 (^223^Ra, half-life 11.4 days); and thorium-227 (^227^Th, half-life 18.7 days) (1). Lead-212 (^212^Pb, half-life 10.64 h) is a β-emitter; however, it generates the daughter nuclides bismuth-212 (^212^Bi) and polonium-212 (^212^Po), which are short-lived α-particle emitters (10). ^223^Ra dichloride was the first and is still the only approved TAT (11, 12), and is approved for use in metastatic castration-resistant prostate cancer (mCRPC) with bone metastases (13, 14). ^223^Ra dichloride acts as a calcium mimetic and is preferentially taken up in osteoblastic bone metastases (15, 16); it cannot currently be complexed to targeting moieties, although recent developments have shown promise (14, 17). Most other TATs, like targeted thorium conjugates (TTCs) or targeted actinium conjugates, use isotopes chelated to various targeting moieties. This enables delivery to a wide range of tumors (14), extending the clinical application of radionuclides.

## 2. Targeted thorium conjugates and their mode of action

^227^Th, the progenitor of ^223^Ra, can be used in TTCs, comprised of the ^227^Th α-emitting radionuclide, a chelator such as octadentate 3,2-hydroxypyridinone (3,2-HOPO), and a tumor-targeting moiety (13, 14). TTCs enable selective delivery of ^227^Th to tumors by targeting antigens expressed in cancer tissues but absent or at low levels in normal tissues (2). For a therapeutic window, TTC characteristics must allow for efficient delivery, accumulation and retention in tumors, while sparing nearby healthy tissue (14). Cytotoxicity results from the induction of clustered double-stranded DNA breaks, followed by subsequent G2/M phase cell cycle arrest and apoptosis (14). Immunogenic cell death has also been demonstrated, occurring *via* increased tumor infiltration by CD8+ T cells (5, 14). The activity of TTCs is not reliant on cellular internalization of ^227^Th, given the α-particle path length of 20–100 μM (2–10 cell diameters) in tissue, a property which may overcome heterogeneous antigen expression (14).

The relatively long half-life of ^227^Th (18.7 days) compared with other radionuclides in current use for TAT (1) highlights the need to identify appropriate targeting moieties that complement the properties of ^227^Th. For example, while typically longer than that of small molecules, the half-lives of antibodies used as therapeutic agents vary considerably (6–32 days) (17–19), suggesting that some may not be suitable for delivery of a radionuclide with a longer half-life. For TTCs, while it may be preferable to select antibodies with comparable half-lives to ^227^Th, data are not yet available as to whether this would be necessary for therapeutic efficacy.

When the ^227^Th component of a TTC decays, recoil energy releases the daughter radionuclide ^223^Ra from the chelator (14). Whilst data on the safety and biodistribution of ^223^Ra released from TTCs are not available, ^223^Ra is well tolerated when it is used as a treatment (20) and it is rapidly cleared from plasma into the small bowel and excreted (21). Furthermore, the amount of ^223^Ra released from a TTC will be much smaller than that of a therapeutic dose of ^223^Ra. Daughter radionuclides of ^227^Th that lie downstream of ^223^Ra in the decay cascade have very short half-lives (14) and have no clinical consequence, as indicated by the good tolerability of ^223^Ra as a cancer therapeutic (20).

## 3. TTCs in cancer

**FIGURE 1:**
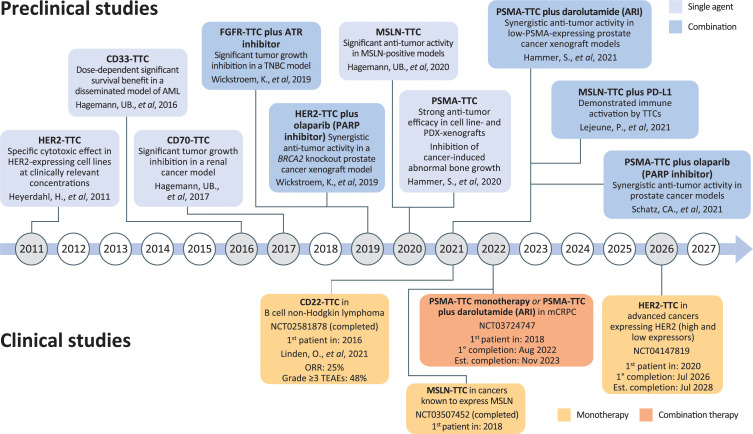
Timeline of TTC development. ARI, androgen receptor inhibitor; CD, cluster of differentiation; FGFR, fibroblast growth factor receptor; HER2, human epidermal growth factor receptor-2; MSLN, mesothelin; PARP, poly (ADP)-ribose polymerase; PD-L1, programmed death-ligand 1; PDX, patient-derived xenograft; PSMA, prostate-specific membrane antigen; TTC, targeted thorium conjugate; AML, acute myeloid leukemia; ATR, ataxia telangiectasia and rad3-related protein; mCRPC, metastatic castration-resistant prostate cancer; ORR, overall response rate; TNBC, triple-negative breast cancer; TEAE, treatment-emergent adverse event.

Pre-clinical and clinical studies of TTCs have included several tumor types expressing a range of different cancer-related antigens (Figure 1).

### 3.1. Hematological cancers

Initial Pre-clinical studies focusing on hematological cancers, targeting CD22 or CD33 in lymphoma and acute myeloid leukemia (AML), respectively, demonstrated promising anti-tumor activity (14, 22). Furthermore, CD22-TTC (BAY 1862864) has been investigated in a Phase 1 study in patients with CD22-positive relapsed/refractory B-cell non-Hodgkin lymphoma (23). In this setting, CD22-TTC was safe, with the most common grade ≥3 adverse events being neutropenia, thrombocytopenia, and leukopenia (23). Maximum ^227^Th blood concentrations increased proportionally to the dose administered and stability of CD22-TTC in the blood was demonstrated (23). The overall objective response rate (ORR) was 24% (5/21 patients: 1 complete and 4 partial responses), with the highest ORR seen in patients with relapsed low-grade lymphomas [3/10 patients (30%)] (23).

### 3.2. Renal cell cancer

CD27, part of the tumor necrosis factor receptor superfamily, plays a vital role in T- and B-cell co-stimulation (24). Physiological expression of CD70, the natural ligand of CD27, is transient and restricted to activated immune cells (24). However, CD70 dysregulation and overexpression has been observed in several cancers (25–28), where it may play a role in tumor progression and immunosuppression (29). Therefore, CD70-TTCs have the potential to both eliminate cancer cells and modulate immune responses. A CD70-TTC has been shown to reduce cell viability in renal cancer cell lines and significantly inhibit tumor growth in a renal cancer xenograft model (25).

### 3.3. Breast cancer

Approximately 25–30% of breast cancers overexpress human epidermal growth factor receptor-2 (HER2), which is associated with more aggressive disease (30). Intrinsic and acquired resistance to HER2-targeting antibodies or antibody drug conjugates (ADC) necessitates development of novel therapies (31, 32). A HER2-TTC, utilizing the HER2 antibody trastuzumab (^227^Th-trastuzumab), showed significant dose-dependent anti-tumor effects in HER2-expressing breast cancer xenografts (33, 34). Moreover, when ^227^Th-trastuzumab was compared with lutetium-177 (^177^Lu; a β-particle emitter) complexed with trastuzumab, in a similar xenograft study, each radionuclide conjugate had significant anti-tumor effects and increased survival, although efficacy was higher with ^227^Th-trastuzumab than with ^177^Lu-trastuzumab. However, ^177^Lu-trastuzumab had a superior therapeutic index (34). Additionally, clinically relevant concentrations of ^227^Th-trastuzumab induced cytotoxic effects in HER2-expressing breast cancer cell lines (35).

Initial HER2-targeted agents were ineffective against HER2-low breast cancer (36). However, the ADC trastuzumab deruxtecan recently demonstrated efficacy in this setting (37). Notably, HER2-TTC has been shown to inhibit tumor growth in HER2-low colorectal cancer (CRC) xenografts (9), highlighting its potential as an alternative treatment option for HER2-low cancers. Furthermore, a Phase I trial of a HER2-TTC is ongoing in advanced HER2-expressing cancers: HER2-high and low expression in breast, gastric/gastroesophageal and other tumors (38).

Fibroblast growth factor receptor 2 (FGFR2) is also a promising target for TTCs, with amplifications in FGFR2 observed in a subset of triple-negative breast cancers (TNBCs) (39–41). Elevated FGFR2 is associated with an aggressive cancer phenotype and resistance to targeted therapy (39, 42), making FGFR2-TTCs an attractive therapeutic option. Indeed, in a human TNBC xenograft model, single-dose FGFR2-TTC reduced tumor growth and was well tolerated (43).

### 3.4. Gastric cancer

HER2 is overexpressed in over 20% of all gastric cancers and is a valid therapeutic target in this setting (44, 45). HER2-TTC was associated with potent target-mediated cytotoxicity in various cancer cell lines, including gastric cancer cell lines, expressing different levels of HER2 (46).

FGFR2 is also a potential target for TTCs, with some gastric cancers overexpressing the protein (47, 48). In gastric cancer xenograft models, tumor growth was inhibited after a single dose of FGFR2-TTC (48).

### 3.5. Colorectal cancer (CRC)

Next-generation sequencing identified *FGFR2* aberrations in a subset (1.4%) of patients with CRC (49) and FGFR2 expression has been seen in 2.9% of patients with CRC (50), indicating some patients may benefit from therapeutic targeting of this protein. In support of this, single-dose FGFR2-TTC inhibited tumor growth in a xenograft model of CRC (48).

HER2-TTC has also been evaluated in CRC models in combination with a poly (ADP)-ribose polymerase (PARP) inhibitor, which is discussed later in this review (9).

### 3.6. Mesothelioma

Mesothelioma is a rare malignant growth of mesothelial cells, occurring in lining layers of the viscera, e.g., pleura, peritoneum and pericardium (51). Mesothelin (MSLN) mediates cellular adhesion and is normally only expressed in mesothelial cells; however, when dysregulated in cancer, MSLN promotes proliferation, migration and invasion, making it an attractive target for TTC-based therapy (52–55). MSLN-TTC has shown potent cytotoxic effects in MSLN-positive cancer cell lines (including mesothelioma) and, when used in single- or multiple-dose regimens in cell line- and patient-derived xenograft models, the conjugate had significant anti-tumor activity and was well tolerated (56). Furthermore, MSLN-TTC prolonged survival in a disseminated lung cancer model in mice (56).

A first-in-human Phase I study of MSLN-TTC in patients with advanced cancer (mesothelioma, as well as MSLN-positive recurrent serous ovarian cancer and pancreatic adenocarcinoma) was completed in the first half of 2022 (57); results are being analyzed for future publication.

### 3.7. Ovarian cancer

Mesothelin-targeted thorium conjugate has been investigated in MSLN-positive ovarian cancer models, with significant anti-tumor activity seen when MSLN-TTC was used in single-dose regimens in cell line-derived xenografts and single- and multiple-dose regimens in patient-derived xenografts (56). Data from the aforementioned first-in-human study of MSLN-TTC in patients with advanced cancer, including ovarian cancer, are awaited with interest.

Pre-clinical studies have also explored the potential for HER2-TTCs in HER2-positive forms. ^227^Th-trastuzumab demonstrated cytotoxic effects in HER2-expressing ovarian cancer cell lines when used at clinically relevant concentrations (35). Furthermore, in HER2-positive ovarian cancer xenograft models, ^227^Th-trastuzumab delayed tumor growth and was associated with survival benefit vs. unlabeled trastuzumab (58, 59) or ^177^Lu-trastuzumab (at the same absorbed radiation dose to tumor) (59). Notably, fractionation of ^227^Th-trastuzumab dosing in xenograft models reduced toxicity while retaining efficacy, showing that administration schedule is an important consideration for TTCs (60).

### 3.8. Prostate cancer

A TTC targeting prostate-specific membrane antigen (PSMA) has been developed. *In vitro*, the antibody-based PSMA-TTC was rapidly internalized in a target-dependent manner, selectively reduced PSMA-expressing cell viability, and induced double-stranded DNA breaks, cell cycle arrest (G2/M phase), and apoptosis in prostate cancer cells (61). Consistent with this, induction of DNA damage markers and apoptosis was observed with PSMA-TTC in patient-derived xenografts in mice (61). Further *in vivo* data showed PSMA-TTC was associated with delayed tumor growth/tumor regression in PSMA-positive patient- and cell line-derived xenograft models mimicking different prostate cancer stages, including models resistant to standard-of-care anti-androgens (including enzalutamide) (61). This effect was seen with single as well as fractionated dosing (61). In a mouse model replicating prostate cancer bone metastases, PSMA-TTC significantly reduced the growth of tumors in the bone and was associated with changes in tumor-induced bone morphology vs. controls (61).

A Phase I clinical study of PSMA-TTC, either alone or in combination with the novel androgen receptor inhibitor (ARI) darolutamide, in patients with mCRPC is currently ongoing; the primary completion date was August 2022, the estimated completion date is November 2023 (62).

## 4. TTCs in combination with other cancer therapies

Due to the unique mode of action of TTCs, there is a strong rationale for combining these with other cancer therapies, and this has been investigated in several pre-clinical studies.

### 4.1. DNA repair pathway inhibitors

As TTCs induce complex double-stranded DNA breaks (1), it is of interest to combine their use with PARP inhibitors, as PARP-1 and PARP-2 are involved in DNA damage repair (63, 64). *BRCA* mutations have been shown to sensitize cells to PARP inhibition (65, 66), as BRCA proteins are crucial for the repair of double-stranded DNA breaks (63). Indeed, in a *BRCA2*-mutated prostate cancer xenograft model, PSMA-TTC plus the PARP inhibitor olaparib showed more notable anti-tumor activity than PSMA-TTC alone, while olaparib alone showed no activity (67).

Additionally, HER2-TTC has been investigated in parental and *BRCA2* knockout HER2-expressing CRC cell lines and their corresponding xenograft models (9). In cell viability assays, the effect of HER2-TTC plus olaparib was synergistic in *BRCA2* knockout cells vs. additive in parental cells (9). Similarly, when combined with olaparib in *BRCA2*-deficient xenografts, low-dose HER2-TTC resulted in similar tumor growth inhibition to high-dose HER2-TTC alone, with the combination concluded as being synergistic; by contrast, no synergistic effects were seen with the combination in the parental xenograft model (9). These findings support further evaluation of PARP inhibitors in combination with TTCs.

Another protein involved in double-stranded DNA break repair is DNA-dependent protein kinase (DNA-PK), which plays a key role in non-homologous end joining (NHEJ) (68). Loss of DNA-PK makes cells more susceptible to radiation, as NHEJ is important for the repair of DNA double-strand breaks that are induced by ionizing radiation (68). Combining PSMA-TTC with a DNA-PK inhibitor resulted in synergistic anti-proliferative effects in prostate cancer cells (69). The combination was also more effective than PSMA-TCC monotherapy in prostate tumor-bearing mice (69), indicating the clinical potential for this combination.

FGFR2-TTC has been investigated in combination with an inhibitor of the ataxia telangiectasia and rad3-related protein (ATR), an enzyme involved in DNA damage response (43, 70–72). *In vitro*, the combination of FGFR2-TTC plus ATR inhibitor reduced cell viability and increased levels of γH2A.X (an indicator of double-strand DNA breaks) vs. FGFR2-TTC alone, while also reducing FGFR2-TTC-mediated cell cycle arrest (43). *In vivo*, tumor growth was significantly inhibited when the two agents were used in combination at single-agent doses known to have no effect (43). Data from ovarian cancer models studying the MSLN-TTC plus ATR inhibitor combination support these findings (7).

### 4.2. Immune checkpoint inhibitors

Immunostimulatory effects have been shown with radiation, including external beam radiotherapy and α-particle emitters, with the former showing anti-tumor effects when combined with immune checkpoint inhibitors (4, 73–76). These data provide rationale for combining a TTC with an immune checkpoint inhibitor, such as programmed death ligand-1 (PD-L1). MSLN-TTC demonstrated a robust immunostimulatory effect in human cancer cell lines (5). Moreover, in immunocompetent mice bearing implanted murine tumors expressing human MSLN, tumor growth was inhibited by MSLN-TTC and anti-PD-L1 individually, with this benefit enhanced when these agents were used in combination (5). Dendritic cell migration out of tumors and CD8+ T-cell infiltration into tumors was observed when MSLN-TTC was administered as monotherapy, with more extensive T-cell infiltration seen when MSLN-TTC was combined with anti-PD-L1 (5).

### 4.3. ARIs

Although ARIs are a common treatment option for patients with prostate cancer, treatment resistance eventually develops (77). This highlights the need for new therapeutic approaches, such as novel combination treatments or new agents with different mechanisms of action, to overcome this therapeutic barrier.

The ARI darolutamide is approved for non-metastatic CRPC in key markets (78, 79) and more recently for use in combination with docetaxel for metastatic hormone-sensitive prostate cancer in the United States (79). Darolutamide has been shown to induce PSMA expression in prostate cancer cell lines and xenografts (80, 81), providing a rationale for combining the drug with a PSMA-TTC. In prostate cancer xenograft models, darolutamide-mediated increase of PSMA expression facilitated tumor uptake of PSMA-TTC, and darolutamide also impaired PSMA-TTC-mediated induction of DNA damage repair genes (80). Furthermore, the combination of PSMA-TTC plus darolutamide demonstrated synergistic inhibition of tumor growth in xenograft models (80). The tumor inhibitory activity of the combination was also more notable than either agent alone in xenograft models that were either resistant to the ARI enzalutamide (80) or hormone independent (81). These results support clinical investigation of this combination.

## 5. Discussion

^227^Th is one of a number of α-emitters suitable for chelation and conjugation to tumor-targeting moieties and thus has the potential to cover a broad tumor range. Indeed, pre-clinical studies have shown anti-tumor activity of TTCs as monotherapy across a broad range of tumor types, and TTCs targeting HER2, PSMA, MSLN, and CD22 are under investigation in clinical studies. Furthermore, there is a strong rationale and pre-clinical evidence for combining TTCs with other targeted therapies, supporting their clinical evaluation. However, no additional TTC clinical trials are currently planned.

In addition to ^227^Th, various other α-emitters are being explored as conjugates for the treatment of cancer. Those considered to be the most suitable include ^225^Ac, ^211^At, ^213^Bi, and ^212^Pb (the latter being a β-emitter that generates daughter α-emitters) (1, 82), with the most clinical experience being available for ^225^Ac and ^213^Bi (83–90).

The clinical potential of targeted radionuclide therapy is further highlighted by the recent US approval of ^177^Lu-PSMA-617 (a β-emitter conjugated to a small molecule PSMA ligand) for the treatment of mCRPC (91–93). Moreover, promising early clinical data has indicated that targeting PSMA with ^225^Ac *via* a small molecule (84, 94, 95) or an antibody (96) has substantial potential in advanced prostate cancer, including for patients who have received radiotherapeutics utilizing ^177^Lu (97), and suggests feasibility of using different targeted radionuclides sequentially.

In summary, TATs represent an important therapeutic development in oncology and offer promise for addressing unmet medical needs for patients, such as resistance to established therapies.

## Author contributions

VW, HH, VJ, and UBH contributed to the conception and design. JK, CAS, AMW, SH, AS, AC, VW, HH, VJ, and UBH contributed to the drafting and revising of the work, and approval of the final version. All authors agreed to be accountable for all aspects of the respective work.
